# Zebrafish as a Model for Drug Screening in Genetic Kidney Diseases

**DOI:** 10.3389/fped.2018.00183

**Published:** 2018-06-28

**Authors:** Jochen Gehrig, Gunjan Pandey, Jens H. Westhoff

**Affiliations:** ^1^Acquifer is a Division of Ditabis, Digital Biomedical Imaging Systems AG, Pforzheim, Germany; ^2^Department of Pediatrics I, University Children's Hospital Heidelberg, Heidelberg, Germany

**Keywords:** zebrafish, drug screening, compound screening, genetic kidney disease, high-throughput, high-content, automated microscopy

## Abstract

Genetic disorders account for a wide range of renal diseases emerging during childhood and adolescence. Due to the utilization of modern biochemical and biomedical techniques, the number of identified disease-associated genes is increasing rapidly. Modeling of congenital human disease in animals is key to our understanding of the biological mechanism underlying pathological processes and thus developing novel potential treatment options. The zebrafish (*Danio rerio*) has been established as a versatile small vertebrate organism that is widely used for studying human inherited diseases. Genetic accessibility in combination with elegant experimental methods in zebrafish permit modeling of human genetic diseases and dissecting the perturbation of underlying cellular networks and physiological processes. Beyond its utility for genetic analysis and pathophysiological and mechanistic studies, zebrafish embryos, and larvae are amenable for phenotypic screening approaches employing high-content and high-throughput experiments using automated microscopy. This includes large-scale chemical screening experiments using genetic models for searching for disease-modulating compounds. Phenotype-based approaches of drug discovery have been successfully performed in diverse zebrafish-based screening applications with various phenotypic readouts. As a result, these can lead to the identification of candidate substances that are further examined in preclinical and clinical trials. In this review, we discuss zebrafish models for inherited kidney disease as well as requirements and considerations for the technical realization of drug screening experiments in zebrafish.

## Introduction

Modern genetic diagnostics allow the rapid discovery of human disease-associated mutations. Moreover, human genetic disorders can often be mimicked in animal models that can be exploited in large-scale chemical investigations for the search of modifiers of disease-associated phenotypes and potentially therapeutic compounds. The zebrafish (*Danio rerio*) has become an increasingly accepted vertebrate model organism for biomedical research (1, 2).

Despite being a member of the teleost class of fish species, there is great homology in development as well as cell- and organ-specific structural and physiological properties between zebrafish and humans. Furthermore, even with the evolutionary distance, > 80% of human disease-associated genes have orthologs in the zebrafish genome (3). The embryonic and larval characteristics of zebrafish include small size, *ex utero* development, optical transparency, and rapidity of organogenesis. In combination with the high fecundity of adult zebrafish and a relatively simple and cost-effective animal husbandry, this enables large-scale *in vivo* investigations. The zebrafish genome has been completely sequenced, thus facilitating genetic and genomic analysis and manipulation (3, 4). For instance, reverse genetics allow for precise investigation of associated phenotypes, by e.g., transient gene knockdown using antisense morpholino oligos or by genome-editing technologies like the CRISPR/Cas9 system (5, 6).

Due to the simplicity of the pronephros that can be readily studied in embryonic and larval stages, the zebrafish is an applicable experimental model system for the analysis of renal development and disease (7). The pronephros, as the earliest nephric stage, contains two nephrons sharing numerous genetic, structural, and functional aspects with the mammalian nephron (8). Phenotypic changes upon genetic alterations can be easily analyzed within intact live animals (9). Large-scale mutagenesis screens have identified various mutants affecting kidney development allowing the exploration of genetic and molecular mechanisms underlying pronephros development and function (10, 11). Moreover, reverse genetics approaches enable researchers to specifically alter orthologous genetic elements potentially associated with human disease. To date, major fields of research where such zebrafish models are being employed include glomerular (i.e., podocytopathies) and cystic renal disorders (i.e., ciliopathies).

Phenotype-based screening for drug discovery applications is increasingly employed in biomedical and pharmaceutical research. In contrast to target-based screening, phenotype-based approaches do not require exact knowledge of the therapeutic target (7). In addition, whole-organism *in vivo* approaches have the advantage that they can unravel toxic and other side-effects of drugs at a very early stage of the study. Over the last years, due to the versatility and power of the model, the zebrafish has emerged as the main vertebrate model system for high-throughput and high-content chemical screening experiments and large-scale phenotypic scoring (12, 13). Clear and scalable readouts for *in vivo* large-scale experiments can be readily established and a plethora of mutant and transgenic models expressing fluorescent proteins driven by tissue-specific promoters is available. In combination with automation technologies and dedicated sample handling workflows, this has led to various biomedical screening assays in fields such as genetics (14, 15), toxicology (16, 17), immunology and infection biology (18, 19), cardiovascular research (20, 21), drug discovery and safety (12, 13, 22, 23), personalized medicine (24), non-coding-genome analysis (25) as well as behavioral analysis (26, 27). Notably, several compounds that were identified in the zebrafish model have made it to preclinical and clinical trials, including new substance classes and repurposed drugs (12). For instance, in a chemical genetic screen testing 2.480 compounds, prostaglandin E2 (PGE2) was identified as an evolutionarily conserved regulator of hematopoietic stem cell (HSC) number in zebrafish embryos (28). Based on these results, a chemical derivative of PGE2 (Prohema), has been developed with the aim of improving the efficiency of HSC transplants using umbilical cord blood. Prohema has meanwhile advanced to Phase II clinical trials.

## Genetic kidney diseases in zebrafish

Genetic kidney diseases can affect all parts of the kidney and its functions. To date, mutations in more than 150 genes have been identified that cause genetic human kidney diseases such as alterations of kidney development or specific glomerular and tubular diseases (29). Nephrogenesis in vertebrates is an intricate process that includes the successive formation of up to three kidneys depending on the species position in the phylogenetic tree: the pronephros, mesonephros, and metanephros (30). There is an increasing complexity with each successive kidney developing, however the structure and function of the basic renal units, the nephrons, remains largely unvaried across vertebrates (31).

**Figure 1:**
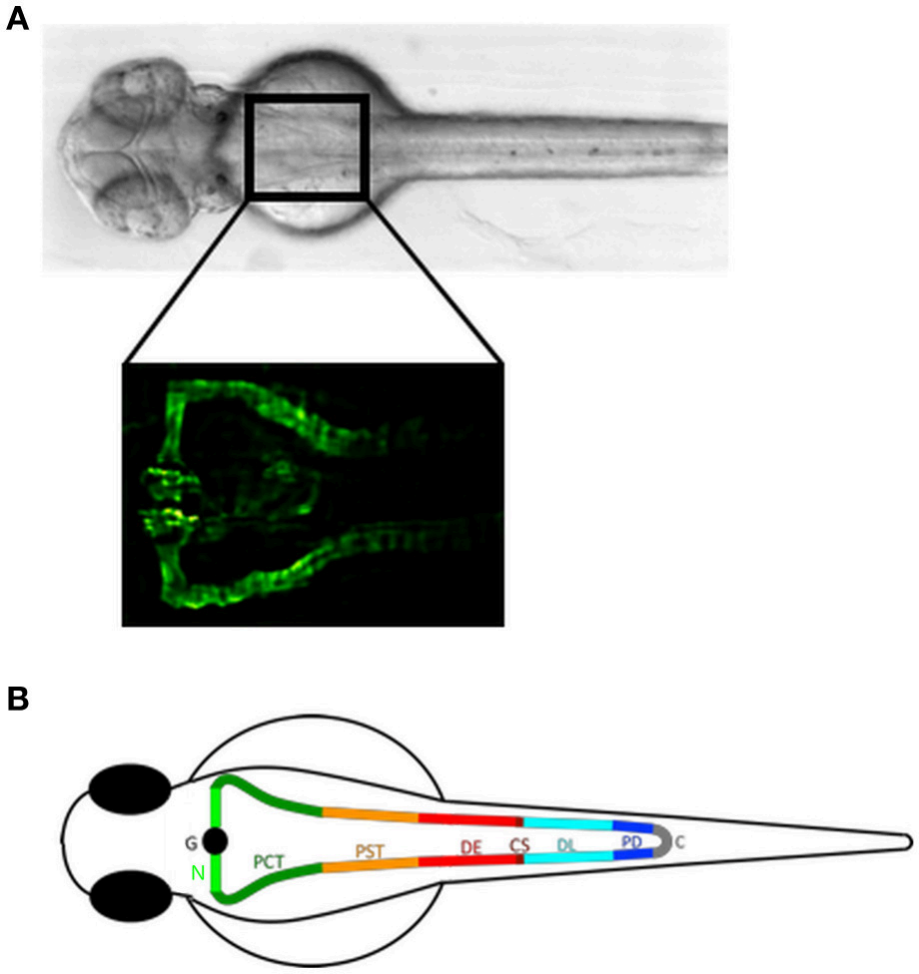
The zebrafish pronephros: anatomical position and segmental organization. **(A)** Brightfield dorsal view of a 2 day post fertilization (dpf) zebrafish embryo (upper panel). The rectangle in the anterior trunk indicates the location of the proximal pronephric structures with a fused glomerulus at the midline that connects to the segmented pronephric tubules as labeled in the *Tg(wt1b:egfp)* zebrafish line by GFP expression (lower panel). **(B)** Schematic illustration of a zebrafish pronephros showing segmental organization of each nephron into glomerulus (G), neck (N), proximal convoluted tubule (PCT), proximal straight tubule (PST), distal early (DE), corpuscle of Stannius (CS), distal late (DL), and pronephric duct (PD) that fuse to the cloaca (C). Adapted from Wingert and Davidson (34).

In zebrafish, major vertebrate organ systems form within a few days after fertilization (32). The zebrafish pronephros is functional by 48 hpf (hours post fertilization) accomplishing the functions of blood filtration and osmoregulation (11, 33). It consists of two nephrons with a fused glomerulus at the midline (Figure 1A) (24). The tubular system consisting of the pronephric proximal and distal tubule and pronephric duct contains segment-specific conserved structural and physiological properties and spatio-temporal gene-expression patterns that are homologous to the human kidney (Figure 1B) (8, 35, 36). The zebrafish glomerulus is endowed with podocytes with extended and interdigitating foot processes and fenestrated endothelial cells forming a functional glomerular filtration barrier, analogous to the metanephric glomerulus of higher vertebrates (11).

At present, zebrafish is predominantly used as a genetic model for normal and abnormal kidney development, for hereditary glomerulopathies (i.e., podocytopathies) and for the study of ciliopathy-associated human cystic kidney diseases. These encompass polycystic kidney diseases and diseases of the nephronophthisis/medullary cystic kidney disease complex including more complex ciliopathies such as Joubert Syndrome, Meckel-Gruber Syndrome, and Bardet-Biedl-Syndrome (37). In this review, we focus on glomerulopathies and cystic kidney diseases.

## Zebrafish as a model for human hereditary glomerulopathies

Zebrafish models can recapitulate human genetic glomerulopathies, i.e., a variety of podocytopathies that clinically often manifest by steroid-resistant nephrotic syndrome (SRNS) due to podocyte foot process effacement. SRNS is mostly therapy-resistant and leads to end-stage renal disease (ESRD) within a few years of onset. A growing number of SRNS-causing mutations have been identified. For example, mutations in *NPHS1*, encoding Nephrin, cause congenital nephrotic syndrome of the Finnish type. Morpholino knockdown of *nphs1* in zebrafish results in edema and loss of slit-diaphragms with abnormal podocyte foot processes (38, 39). Mutations in *WT1* (Wilms' tumor gene 1) have been associated with syndromic disorders such as Denys-Drash syndrome and Frasier syndrome, but also with diffuse mesangial sclerosis and early-onset isolated nephrotic syndrome (40). In zebrafish, knockdown of *wt1a* results in defects in podocyte development leading to glomerular injury and nephrosis (41). Mutations in *NPHS2*, Podocin, are the most relevant cause of autosomal-recessive SRNS of childhood. Zebrafish *nphs2* morphants display pronephric glomerular hypoplasia with pericardial edema and ultrastructural glomerular damage of the filtration barrier (38, 39). Mutations in *PLCE1* (Nephrocystin-3, NPHS3) have been identified in patients with SRNS and disease onset in the first year of life with a rapid progression to ESRD (42, 43). Zebrafish *plce1* morphants display an impairment of the kidney filtration barrier as measured by tubular uptake of filtered 500 kDa fluorescent dextran, accompanied by edema, and severe disorganization of slit diaphragms (43). Other rare human mutations that were mimicked in zebrafish include *ADCK4* (AarF domain containing kinase 4 gene) (44), *KANK1* (kidney ankyrin repeat-containing protein 1), *KANK2, KANK4* (45), *CRB2* (Crumbs homolog 2) (46), *NUP107* (Nuclear Pore Complex Subunit 107) (47), and *ARHGDIA* (48).

Whereas in many studies of genetic glomerulopathies the zebrafish has been used to model human disease-associated phenotypes, disruption of the glomerular filtration barrier can only be visualized by ultrastructural techniques like super-resolution or electron microscopy that are not compatible with large-scale chemical screens. Edema formation in zebrafish embryos can indirectly report glomerular barrier impairments; however, despite being easily observed in brightfield microscopy it is not exclusively linked to renal impairment (49). This restricts its value as a phenotypic readout parameter in chemical kidney screens. Functional assessment of glomerular filtration and barrier integrity can be achieved by monitoring the temporal reporter activity after microinjection of fluorescently labeled inulin (50) or dextrans of different molecular weight (51–53) into the vascular system; however, this method is laborious and incompatible with extensive screening experiments. Additionally, a transgenic zebrafish that expresses GFP (green fluorescent protein)-tagged vitamin D-binding protein (VDBP), which acts as a tracer for proteinuria, has been reported (54) and may serve as an attractive alternative for high-content and high-throughput screening (53).

## Zebrafish as a model for human cystic kidney diseases

Cystic diseases of the kidney are frequent monogenic disorders in humans (55, 56), with primary cilia dysfunction being the unifying cellular mechanism leading to most if not all cystic kidney diseases (56–58). Mutations in a variety of genes encoding the primary cilia/centrosome complex cause ciliopathies often associated with the development of renal cysts, in both human and zebrafish (59–64). However, it must be noted that cilia in the zebrafish pronephros are motile, whereas human renal cilia are thought to be non-motile (65, 66), suggesting a potential contribution of lack of fluid dynamics to cyst formation in the zebrafish model.

Mutations in polycystin-1 and polycystin-2 are responsible for autosomal dominant polycystic kidney disease (ADPKD), the most common human congenital renal disorder (67). In zebrafish, polycystin-2 morpholino knockdown or mutation of orthologous *pkd* genes induces kidney cysts, hydrocephalus, left/right asymmetry defects, and strong dorsal axis curvature (63, 68, 69). Autosomal recessive polycystic kidney disease (ARPKD) usually manifests perinatally or in childhood. In addition to *PKHD1* (polycystic kidney and hepatic disease 1), mutations in *DZIP1L* (DAZ interacting zinc finger protein 1 like) have recently been associated with ARPKD (70, 71). Other than *dzip1l* morphants and *dzip1l* CRISPR mutants, Lu et al. injected the *dzip1l* translation-blocking morpholino into *Tg(wt1b:egfp)* transgenic embryos expressing GFP under Wilms' tumor suppressor *(wt1b)* promoter in the pronephros (72), allowing for *in vivo* fluorescence imaging of the cystic pronephros.

Nephronophthisis (NPHP), an autosomal recessive cystic kidney disease, represents the most frequent genetic cause of ESRD in the first three decades of life (55). Nephronophthisis can be accompanied by anomalies in other organs, such as cerebellar vermis hypoplasia, laterality defects, intellectual disability, shortening of bones, retinal degeneration, and hepatobiliary disease (56). These features are represented in a variety of syndromes, including Senior–Løken syndrome, Joubert syndrome, Bardet–Biedl syndrome, and Jeune asphyxiating thoracic dystrophy (73, 74). To date, NPHP-causing mutations have been identified in more than 20 genes (56). Morpholino knockdown in zebrafish has been performed for *nphp2* to *6* (75–83) and *nphp11* (84). Zebrafish mutants for *arl13b/sco*^*hi*459^ (85), *ahi1*^*lri*46^ (86), and *cc2d2a* (87) develop pronephric cysts to varying degrees and serve as models for Joubert syndrome-related disease.

Intraflagellar transport (IFT) proteins are essential for the development and maintenance of motile and sensory cilia and localize to the cilium, basal body, and/or centrosome (88). Several zebrafish *ift* mutants demonstrating renal cysts were identified by forward genetic screens (63, 89). *IFT80* mutations underlie a subset of Jeune asphyxiating thoracic dystrophy cases, of which 20% are associated with kidney abnormalities including renal cysts (90). *Ift80* morphants show a dose-dependent phenotype with strong body curvature, large kidney cyst, and pericardial edema. *IFT172* mutations were initially reported in Jeune and Mainzer-Saldino syndromes, but have also been observed in patients with Bardet-Biedl syndrome (91, 92). Zebrafish mutants and morphants for *ift172* resemble these phenotypes, with renal cysts readily detectable in brightfield images (91). In our work, we have shown that morpholino knockdown of *ift80* and *ift172* in *Tg(wt1b:egfp)* with fluorescently labeled kidney structures allow for visualization of pronephric cysts, providing a model system for large-scale chemical screening studies to identify chemical modifiers of cyst formation (93).

## Chemical screening in zebrafish—technical aspects and considerations

The zebrafish can cost-effectively bridge the gap between high-throughput experimentation in cellular models lacking physiological context and low-throughput models such as rodents that are closer to human biology (12, 13, 23, 94). The optically transparent embryos and larvae fit into wells of microtiter plates rendering them amenable for automated microscopy applications using existing screening technologies (12, 13, 17, 23). Due to accessibility to genetic manipulation (37, 95), a plethora of zebrafish transgenic and mutant lines has been generated (www.zfin.org, www.ezrc.kit.edu, www.zebrafish.org) (96), complemented by transient labeling, knockdown and genome-editing techniques (97). This provides an extraordinary rich toolkit to model and visualize the biological processes underlying development and disease. Several transgenic lines labeling pronephric structures such as podocytes (98, 99), tubules, and ducts (98, 100–106) or both (72) have been established. In combination with genetic alteration, either by transient gene knockdown using antisense morpholino oligos or by genome-editing technologies, these lines can mimic e.g., ARPKD (71), ADPKD (107), and other cystic kidney diseases (93, 108) and enable *in vivo* fluorescence microscopy of the diseased pronephros. Although controversies exist regarding the use of morphants generated by morpholino knockdown (109–113), they remain a valuable tool in altering target gene expression, provided that all mandatory control experiments to validate the observed disease-associated phenotypes have been conducted (110, 111).

To our knowledge, large-scale screening experiments evaluating fluorescent pronephric phenotypes in models of genetic kidney diseases have not been demonstrated. In a chemical modifier screen using a custom library of 115 compounds in *pkd2*^*hi*4166^ and *ift172*^*hi*2211^ mutants displaying renal cysts (114), morphological parameters such as body axis curvature and/or laterality defects were scored. Histone deacetylase inhibitors Tricostatin A and valproic acid attenuated these phenotypes, and cyst size-reducing effects were confirmed in secondary assays. Additionally, a chemical screen of ~2,000 compounds identified histone deacetylase inhibitors to expand the pool of embryonic renal progenitor cells (115), a mechanism presumably involved in regeneration following acute kidney injury.

In combination with automated microscopy (116), zebrafish disease model systems allow performing large-scale phenotypic whole organism screening assays (117, 118). Phenotypic readouts encompass survival rates, overall morphology, physiological parameters, cell- and tissue-specific phenotypes, reporter gene expression patterns, and behavioral phenotypes (25, 27, 93, 119–123). Phenotypic screens within the context of a live vertebrate provide significant advantage over classical target-based *in vitro* assays as they do not require a priori knowledge about biochemical pathways affected, thus allowing unbiased identification of drug candidates or toxicological effects of substances. Furthermore, they intrinsically involve potential contributions of biodistribution, metabolism, and pharmacokinetics (1, 12, 13, 17, 95, 124).

Prior to large-scale zebrafish experiments careful planning and preparatory experiments are required to generate robust protocols and tailor conditions toward desired readouts in screening compatible disease models. In brief, handling of thousands of embryos causes logistical challenges, thus protocols for animal husbandry, micromanipulation, embryo culture, and treatment (e.g., anesthesia), and sample handling must be established. When image-based assays are carried out the usage of pigmentation mutant strains (e.g., casper line) or chemical treatment (e.g., 1-phenyl-2-thiourea, PTU) to block pigment formation is often necessary to adequately visualize internal structures. Moreover, to minimize false positive and negative results, non-specific developmental toxicity or off-target effects, control experiments must be carried out to titrate required compound concentration ranges as well as the treatment period during embryonic development. Importantly, controls should also be continuously carried out as a reference readout to benchmark observed phenotypic effects and normalize experimental variation. Finally, image acquisition routines must be balanced with analysis needs to ensure effective and robust scoring of phenotypic alterations.

**Figure 2:**
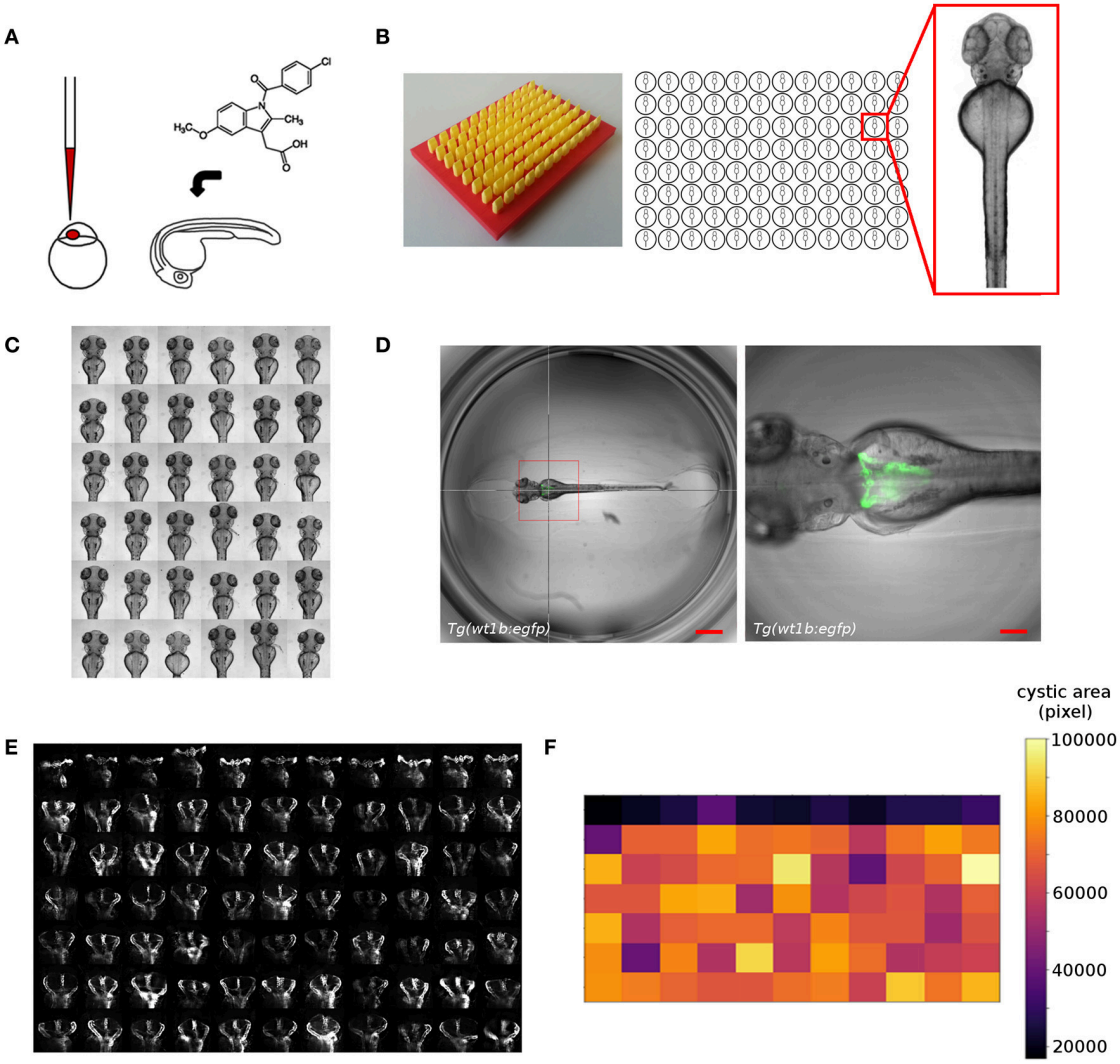
Overview of screening workflows for organ specific phenotypic screening in zebrafish. Shown are examples from our screening work that illustrate the automatic acquisition of higher resolution datasets of embryonic kidneys in zebrafish embryos. **(A)** Experimental manipulation of embryos prior to mounting and automated imaging such as microinjection or compound treatment. **(B)** Mounting of zebrafish embryos in agarose coated microtiter plates generated using 3D printed orientation tools. Agarose layers contain cavities allowing for consistent alignment and orientation of specimen. **(C)** Automated acquisition of standardized views (e.g., dorsal) of zebrafish embryos arrayed in microtiter plates. **(D)** Automated acquisition of multidimensional image datasets using smart imaging techniques. Pronephric areas of the *Tg(wt1b:egfp)* zebrafish transgenic line are detected in low resolution datasets using image processing tools and are subsequently imaged at higher resolution. The hair cross indicates the detected position and the bounding box the field of view in subsequent higher resolution imaging. Scale bars indicate 600 μm (left panel) or 150 μm (right panel). **(E)** Detailed visualization of kidney regions enabling scoring of kidney phenotypes. Shown are wildtype (first row) or cystic (other rows) kidneys of 72 hpf *Tg(wt1b:egfp)* embryos. **(F)** Automated quantitative analysis and phenotypic scoring using image processing techniques. Heatmap shows quantitative measurements of cystic areas as shown in **(E)**. Figure panels are taken or modified from Westhoff et al. (93), Wittbrodt et al. (125), Pandey et al. (unpublished), and www.acquifer.de.

Despite its amenability to large-scale experimentation, the full exploitation of the zebrafish model in screening assays is often hampered. While sample manipulation can be scaled efficiently, large-scale imaging, and phenotypic scoring remains challenging as available screening methodologies are usually optimized for *in vitro* assays (Figure 2A) (126). In comparison to cellular models, zebrafish embryos are large three-dimensional objects of complex morphology leading to random orientation of embryos within wells of microtiter plates (126, 127). This can obscure the view on target structures and leads to the generation of non-standardized image data. Therefore, novel sample preparations or automation strategies are needed, as it is unfeasible to upscale classical zebrafish mounting techniques. In our work, we developed orientation tools allowing the generation of agarose cavities within wells of microtiter plates for consistent positioning and orientation of zebrafish embryos (Figure 2B) (25, 93, 125). This enables the automated acquisition of consistent views of 48–96 hpf zebrafish larvae in large-scale screening scenarios (Figure 2C). For instance, we employed that methodology for imaging of embryonic kidneys in automated large-scale microscopy assays to score for morphological alterations of the pronephros upon compound exposure (93, 125, 128) (Westhoff et al. unpublished data) or capture phenotypic changes in cystic kidney disease models (129) (Pandey et al., unpublished data). Other more complex technical solutions employ microfluidic systems that combine automated detection and rotational orientation within glass capillaries followed by microscopic imaging (127, 130).

To date, the vast majority of zebrafish screens employ low magnification to capture the entire zebrafish embryo body, followed by subsequent analysis of phenotypic changes (13, 131). However, this significantly attenuates the power of using a fully developed live vertebrate embryo as *in vivo* visualization of morphological, physiological or genetic events on the cell- or tissue-specific level is hampered. The widespread usage of low resolution assays is largely due to the impracticality of positioning the regions of interest (ROI) within the small field of views of higher magnification objectives in combination with fixed scan-field coordinates of automated microscopes. Additionally, the spatiotemporal location of ROIs within the embryo body might be variable or unpredictable. To overcome that limitation, technologies have been developed that allow the automatic centering of ROIs in front of objective lenses of microscopes. Microfluidic systems (127, 130) can fulfill that requirement but usually require rather complex setups and are potentially challenged when overall morphological changes occur, or developmental and disease-associated processes are observed in time-lapse experiments. Several automated microscopy solutions for microplate-based screening have been reported that allow to teach or detect the position of ROIs followed by automatic centering and multidimensional image acquisition (126, 132–134). This can be an expert operator manually selecting target structures for subsequent automated imaging or more advanced methods employing automatic detection by image processing. These automated smart imaging approaches are based on interfacing the imaging device with external software tools that automatically detect coordinates of features of interest and send back machine commands containing instructions for re-centering, higher resolution imaging, or tracking of target structures. While several solutions have been reported, their application is usually restricted to cellular models and they are often characterized by a high level of complexity requiring expert knowledge in image processing, general programming and hard- and software interfacing. Therefore, to enable a widespread usage of such toolsets novel developments are needed that provide a simplified access to biomedical researchers. To this extent, we have developed a robotic microscopy platform (www.acquifer.de) with a smart imaging interface that allows to manually select ROIs, or to use any image processing software to send back human-readable script commands to the imaging device. We utilize this technique to e.g., acquire high-resolution datasets of cystic kidneys in a genetic zebrafish disease model to screen for modifiers of cystogenesis (Figures 2D–F) (129) (Pandey et al., unpublished data).

Due to the wide variety of potential zebrafish screening assays and thus phenotypic readouts there are myriad of potential quantitative descriptors that can be extracted from image-based datasets. This can include fluorescence intensity, morphological descriptors, or dynamic parameters and ranges from simple whole embryo signal intensity to spatiotemporal activity of fluorescent reporters or tissue dynamics and beyond. For instance, advanced segmentation techniques were used in the *Tg(cdh17:egfp)*^*pt*305^ zebrafish line to detect the fluorescently labeled tubular cells of the kidney (105). A full discussion of analysis strategies and development of automated image processing pipelines is beyond the scope of this manuscript (135). However, as post-acquisition analysis strategies are of vital importance for the success of any screening assay, the design of scoring pipelines needs careful consideration at early stages of experimental planning.

## Conclusions

Phenotype-based, cost-effective whole-organism chemical screening in zebrafish offers a variety of advantages including the identification of disease-modifying drugs without knowledge of a validated target, the potential to identify compounds with polypharmacological efficacy, and the simultaneous assessment of compound efficacy, toxicity, biodistribution, and pharmacokinetics within a vertebrate model system. While a growing number of genes are being identified to cause human kidney diseases, therapeutic options to combat these diseases are often absent. Ideally, the use of genetically modified zebrafish mimicking human genetic disorders in conjunction with kidney-specific transgenic reporter lines or in conjunction with fluorescently-labeled functional reporter lines (or other secondary readouts), permit the implementation of chemical screening for disease-modifying substances in the field of genetic kidney diseases.

## Author contributions

All authors listed have made a substantial, direct, and intellectual contribution to the work, and approved it for publication.

### Conflict of interest statement

GP and JG are employees of DITABIS AG, Pforzheim, Germany. The remaining author declares that the research was conducted in the absence of any commercial or financial relationships that could be construed as a potential conflict of interest.

## References

[1] Lieschke GJ Currie PD. Animal models of human disease: zebrafish swim into view. Nat Rev Genet. (2007) 8:353–67. 10.1038/nrg2091 17440532

[2] Dooley K Zon LI. Zebrafish: a model system for the study of human disease. Curr Opin Genet Dev. (2000) 10:252–6. 10.1016/S0959-437X(00)00074-5 10826982

[3] Howe K Clark MD Torroja CF Torrance J Berthelot C Muffato M. The zebrafish reference genome sequence and its relationship to the human genome. Nature (2013) 496:498–503. 10.1038/nature12111 23594743 PMC3703927

[4] Ota S Kawahara A. Zebrafish: a model vertebrate suitable for the analysis of human genetic disorders. Congenit Anom. (2014) 54:8–11. 10.1111/cga.12040 24279334

[5] Cong L Ran FA Cox D Lin S Barretto R Habib N. Multiplex genome engineering using CRISPR/Cas systems. Science (2013) 339:819–23. 10.1126/science.1231143 23287718 PMC3795411

[6] Mali P Yang L Esvelt KM Aach J Guell M DiCarlo JE. RNA-guided human genome engineering via Cas9. Science (2013) 339:823–6. 10.1126/science.1232033 23287722 PMC3712628

[7] Swanhart LM Cosentino CC Diep CQ Davidson AJ de Caestecker M Hukriede NA. Zebrafish kidney development: basic science to translational research. Birth Defects Res C Embryo Today (2011) 93:141–56. 10.1002/bdrc.20209 21671354 PMC3694221

[8] Wingert RA Selleck R Yu J Song HD Chen Z Song A. The cdx genes and retinoic acid control the positioning and segmentation of the zebrafish pronephros. PLoS Genet. (2007) 3:1922–38. 10.1371/journal.pgen.0030189 17953490 PMC2042002

[9] Morales EE Wingert RA. Zebrafish as a model of kidney disease. Results Probl Cell Differ. (2017) 60:55–75. 10.1007/978-3-319-51436-9_3 28409342

[10] Driever W Solnica-Krezel L Schier AF Neuhauss SC Malicki J Stemple DL. A genetic screen for mutations affecting embryogenesis in zebrafish. Development (1996) 123:37–46. 9007227 10.1242/dev.123.1.37

[11] Drummond IA Majumdar A Hentschel H Elger M Solnica-Krezel L Schier AF. Early development of the zebrafish pronephros and analysis of mutations affecting pronephric function. Development (1998) 125:4655–67. 9806915 10.1242/dev.125.23.4655

[12] MacRae CA Peterson RT. Zebrafish as tools for drug discovery. Nat Rev Drug Discov. (2015) 14:721–31. 10.1038/nrd4627 26361349

[13] Rennekamp AJ Peterson RT. 15 years of zebrafish chemical screening. Curr Opin Chem Biol. (2015) 24:58–70. 10.1016/j.cbpa.2014.10.025 25461724 PMC4339096

[14] Shah AN Davey CF Whitebirch AC Miller AC Moens CB. Rapid reverse genetic screening using CRISPR in zebrafish. Nat Methods (2015) 12:535–40. 10.1038/nmeth.3360 25867848 PMC4667794

[15] Dang M Fogley R Zon LI. Identifying novel cancer therapies using chemical genetics and zebrafish. Adv Exp Med Biol. (2016) 916:103–24. 10.1007/978-3-319-30654-4_5 27165351 PMC6022836

[16] Sipes NS Padilla S Knudsen TB. Zebrafish: as an integrative model for twenty-first century toxicity testing. Birth Defects Res C Embryo Today (2011) 93:256–67. 10.1002/bdrc.20214 21932434

[17] Yang L Ho NY Alshut R Legradi J Weiss C Reischl M. Zebrafish embryos as models for embryotoxic and teratological effects of chemicals. Reprod Toxicol. (2009) 28:245–53. 10.1016/j.reprotox.2009.04.013 19406227

[18] Sullivan C Matty MA Jurczyszak D Gabor KA Millard PJ Tobin DM. Infectious disease models in zebrafish. Methods Cell Biol. (2017) 138:101–36. 10.1016/bs.mcb.2016.10.005 28129840

[19] Astin JW Keerthisinghe P Du L Sanderson LE Crosier KE Crosier PS. Innate immune cells and bacterial infection in zebrafish. Methods Cell Biol. (2017) 138:31–60. 10.1016/bs.mcb.2016.08.002 28129850

[20] Kessler M Rottbauer W Just S. Recent progress in the use of zebrafish for novel cardiac drug discovery. Expert Opin Drug Discov. (2015) 10:1231–41. 10.1517/17460441.2015.1078788 26294375

[21] Vazao H Rosa S Barata T Costa R Pitrez PR Honorio I. High-throughput identification of small molecules that affect human embryonic vascular development. Proc Natl Acad Sci USA. (2017) 114:E3022–31. 10.1073/pnas.1617451114 28348206 PMC5393190

[22] Eimon PM Rubinstein AL. The use of *in vivo* zebrafish assays in drug toxicity screening. Expert Opin Drug Metab Toxicol. (2009) 5:393–401. 10.1517/17425250902882128 19368493

[23] Brady CA Rennekamp AJ Peterson RT. Chemical screening in Zebrafish. Methods Mol Biol. (2016) 1451:3–16. 10.1007/978-1-4939-3771-4_1 27464797

[24] Baxendale S van Eeden F Wilkinson R. The power of Zebrafish in personalised medicine. Adv Exp Med Biol. (2017) 1007:179–97. 10.1007/978-3-319-60733-7_10 28840558

[25] Gehrig J Reischl M Kalmar E Ferg M Hadzhiev Y Zaucker A. Automated high-throughput mapping of promoter-enhancer interactions in zebrafish embryos. Nat Methods (2009) 6:911–6. 10.1038/nmeth.1396 19898487

[26] Bruni G Lakhani P Kokel D. Discovering novel neuroactive drugs through high-throughput behavior-based chemical screening in the zebrafish. Front Pharmacol. (2014) 5:153. 10.3389/fphar.2014.00153 25104936 PMC4109429

[27] Rihel J Schier AF. Behavioral screening for neuroactive drugs in zebrafish. Dev Neurobiol. (2012) 72:373–85. 10.1002/dneu.20910 21567979

[28] North TE Goessling W Walkley CR Lengerke C Kopani KR Lord AM. Prostaglandin E2 regulates vertebrate haematopoietic stem cell homeostasis. Nature (2007) 447:1007–11. 10.1038/nature05883 17581586 PMC2775137

[29] Eckardt KU Coresh J Devuyst O Johnson RJ Kottgen A Levey AS. Evolving importance of kidney disease: from subspecialty to global health burden. Lancet (2013) 382:158–69. 10.1016/S0140-6736(13)60439-0 23727165

[30] Drummond IA. Zebrafish kidney development. Methods Cell Biol. (2004) 76:501–30. 10.1016/S0091-679X(04)76023-9 15602890

[31] Desgrange A Cereghini S. Nephron patterning: lessons from Xenopus, Zebrafish, and Mouse studies. Cells (2015) 4:483–99. 10.3390/cells4030483 26378582 PMC4588047

[32] Ebarasi L Oddsson A Hultenby K Betsholtz C Tryggvason K. Zebrafish: a model system for the study of vertebrate renal development, function, and pathophysiology. Curr Opin Nephrol Hypertens. (2011) 20:416–24. 10.1097/MNH.0b013e3283477797 21519251

[33] Drummond IA. The zebrafish pronephros: a genetic system for studies of kidney development. Pediatr Nephrol. (2000) 14:428–35. 10.1007/s004670050788 10805474

[34] Wingert RA Davidson AJ. The zebrafish pronephros: a model to study nephron segmentation. Kidney Int. (2008) 73:1120–7. 10.1038/ki.2008.37 18322540

[35] Anzenberger U Bit-Avragim N Rohr S Rudolph F Dehmel B Willnow TE. Elucidation of megalin/LRP2-dependent endocytic transport processes in the larval zebrafish pronephros. J Cell Sci. (2006) 119(Pt 10):2127–37. 10.1242/jcs.02954 16638803

[36] Nichane M Van Campenhout C Pendeville H Voz ML Bellefroid EJ. The Na+/PO4 cotransporter SLC20A1 gene labels distinct restricted subdomains of the developing pronephros in Xenopus and zebrafish embryos. Gene Expr Patterns (2006) 6:667–72. 10.1016/j.modgep.2006.01.005 16531124

[37] Santoriello C Zon LI. Hooked! Modeling human disease in zebrafish. J Clin Invest. (2012) 122:2337–43. 10.1172/JCI60434 22751109 PMC3386812

[38] Kramer-Zucker AG Wiessner S Jensen AM Drummond IA. Organization of the pronephric filtration apparatus in zebrafish requires nephrin, podocin and the FERM domain protein mosaic eyes. Dev Biol. (2005) 285:316–29. 10.1016/j.ydbio.2005.06.038 16102746 PMC2836015

[39] Fukuyo Y Nakamura T Bubenshchikova E Powell R Tsuji T Janknecht R. Nephrin and podocin functions are highly conserved between the zebrafish pronephros and mammalian metanephros. Mol Med Rep. (2014) 9:457–65. 10.3892/mmr.2013.1844 24337247 PMC3896505

[40] Niaudet P Gubler MC. WT1 and glomerular diseases. Pediatr Nephrol. (2006) 21:1653–60. 10.1007/s00467-006-0208-1 16927106

[41] Hall G Gbadegesin RA Lavin P Wu G Liu Y Oh EC et al. A novel missense mutation of Wilms' Tumor 1 causes autosomal dominant FSGS. J Am Soc Nephrol. (2015) 26:831–43. 10.1681/ASN.2013101053 25145932 PMC4378093

[42] Gbadegesin R Hinkes BG Hoskins BE Vlangos CN Heeringa SF Liu J. Mutations in PLCE1 are a major cause of isolated diffuse mesangial sclerosis (IDMS). Nephrol Dial Transplant. (2008) 23:1291–7. 10.1093/ndt/gfm759 18065803

[43] Hinkes B Wiggins RC Gbadegesin R Vlangos CN Seelow D Nurnberg G. Positional cloning uncovers mutations in PLCE1 responsible for a nephrotic syndrome variant that may be reversible. Nat Genet. (2006) 38:1397–405. 10.1038/ng1918 17086182

[44] Ashraf S Gee HY Woerner S Xie LX Vega-Warner V Lovric S. ADCK4 mutations promote steroid-resistant nephrotic syndrome through CoQ10 biosynthesis disruption. J Clin Invest. (2013) 123:5179–89. 10.1172/JCI69000 24270420 PMC3859425

[45] Gee HY Zhang F Ashraf S Kohl S Sadowski CE Vega-Warner V. KANK deficiency leads to podocyte dysfunction and nephrotic syndrome. J Clin Invest. (2015) 125:2375–84. 10.1172/JCI79504 25961457 PMC4497755

[46] Ebarasi L Ashraf S Bierzynska A Gee HY McCarthy HJ Lovric S. Defects of CRB2 cause steroid-resistant nephrotic syndrome. Am J Hum Genet. (2015) 96:153–61. 10.1016/j.ajhg.2014.11.014 25557779 PMC4289689

[47] Miyake N Tsukaguchi H Koshimizu E Shono A Matsunaga S Shiina M. Biallelic mutations in nuclear pore complex subunit NUP107 cause early-childhood-onset steroid-resistant nephrotic syndrome. Am J Hum Genet. (2015) 97:555–66. 10.1016/j.ajhg.2015.08.013 26411495 PMC4596915

[48] Gee HY Saisawat P Ashraf S Hurd TW Vega-Warner V Fang H. ARHGDIA mutations cause nephrotic syndrome via defective RHO GTPase signaling. J Clin Invest. (2013) 123:3243–53. 10.1172/JCI69134 23867502 PMC3726174

[49] Hill AJ Bello SM Prasch AL Peterson RE Heideman W. Water permeability and TCDD-induced edema in zebrafish early-life stages. Toxicol Sci. (2004) 78:78–87. 10.1093/toxsci/kfh056 14718644

[50] Rider SA Tucker CS del-Pozo J Rose KN MacRae CA Bailey MA. Techniques for the *in vivo* assessment of cardio-renal function in zebrafish (*Danio rerio*) larvae. J Physiol. (2012) 590(Pt 8):1803–9. 10.1113/jphysiol.2011.224352 22331420 PMC3573304

[51] Kotb AM Muller T Xie J Anand-Apte B Endlich K Endlich N. Simultaneous assessment of glomerular filtration and barrier function in live zebrafish. Am J Physiol Renal Physiol. (2014) 307:F1427–34. 10.1152/ajprenal.00029.2014 25298528 PMC4347739

[52] Hentschel DM Park KM Cilenti L Zervos AS Drummond I Bonventre JV. Acute renal failure in zebrafish: a novel system to study a complex disease. Am J Physiol Renal Physiol. (2005) 288:F923–9. 10.1152/ajprenal.00386.2004 15625083

[53] Hanke N King BL Vaske B Haller H Schiffer M. A Fluorescence-based assay for proteinuria screening in larval zebrafish (*Danio rerio*). Zebrafish (2015) 12:372–6. 10.1089/zeb.2015.1093 26125680 PMC4593894

[54] Zhou W Hildebrandt F. Inducible podocyte injury and proteinuria in transgenic zebrafish. J Am Soc Nephrol. (2012). 23:1039–47. 10.1681/ASN.2011080776 22440901 PMC3358760

[55] Hildebrandt F Zhou W. Nephronophthisis-associated ciliopathies. J Am Soc Nephrol. (2007) 18:1855–71. 10.1681/ASN.2006121344 17513324

[56] Arts HH Knoers NV. Current insights into renal ciliopathies: what can genetics teach us? Pediatr Nephrol (2013) 28:863–74. 10.1007/s00467-012-2259-9 22829176 PMC3631122

[57] Hildebrandt F Otto E. Cilia and centrosomes: a unifying pathogenic concept for cystic kidney disease? Nat Rev Genet. (2005) 6:928–40. 10.1038/nrg1727 16341073

[58] Watnick T Germino G. From cilia to cyst. Nat Genet. (2003) 34:355–6. 10.1038/ng0803-355 12923538

[59] Hostetter CL Sullivan-Brown JL Burdine RD. Zebrafish pronephros: a model for understanding cystic kidney disease. Dev Dyn. (2003) 228:514–22. 10.1002/dvdy.10371 14579389

[60] Liu S Lu W Obara T Kuida S Lehoczky J Dewar K. A defect in a novel Nek-family kinase causes cystic kidney disease in the mouse and in zebrafish. Development (2002) 129:5839–46. 10.1242/dev.00173 12421721

[61] Low SH Vasanth S Larson CH Mukherjee S Sharma N Kinter MT. Polycystin-1, STAT6, and P100 function in a pathway that transduces ciliary mechanosensation and is activated in polycystic kidney disease. Dev Cell (2006) 10:57–69. 10.1016/j.devcel.2005.12.005 16399078

[62] Otto EA Schermer B Obara T O'Toole JF Hiller KS Mueller AM. Mutations in INVS encoding inversin cause nephronophthisis type 2, linking renal cystic disease to the function of primary cilia and left-right axis determination. Nat Genet. (2003) 34:413–20. 10.1038/ng1217 12872123 PMC3732175

[63] Sun Z Amsterdam A Pazour GJ Cole DG Miller MS Hopkins N A genetic screen in zebrafish identifies cilia genes as a principal cause of cystic kidney. Development (2004) 131:4085–93. 10.1242/dev.01240 15269167

[64] Tobin JL Beales PL. Restoration of renal function in zebrafish models of ciliopathies. Pediatr Nephrol. (2008) 23:2095–9. 10.1007/s00467-008-0898-7 18604564 PMC7462901

[65] Kramer-Zucker AG Olale F Haycraft CJ Yoder BK Schier AF Drummond IA. Cilia-driven fluid flow in the zebrafish pronephros, brain and Kupffer's vesicle is required for normal organogenesis. Development (2005) 132:1907–21. 10.1242/dev.01772 15790966

[66] Satir P Christensen ST. Structure and function of mammalian cilia. Histochem Cell Biol. (2008) 129:687–93. 10.1007/s00418-008-0416-9 18365235 PMC2386530

[67] Le Corre S Eyre D Drummond IA Modulation of the secretory pathway rescues zebrafish polycystic kidney disease pathology. J Am Soc Nephrol. (2014) 25:1749–59. 10.1681/ASN.2013101060 24627348 PMC4116068

[68] Obara T Mangos S Liu Y Zhao J Wiessner S Kramer-Zucker AG. Polycystin-2 immunolocalization and function in zebrafish. J Am Soc Nephrol. (2006) 17:2706–18. 10.1681/ASN.2006040412 16943304 PMC3698611

[69] Schottenfeld J Sullivan-Brown J Burdine RD. Zebrafish curly up encodes a Pkd2 ortholog that restricts left-side-specific expression of southpaw. Development (2007) 134:1605–15. 10.1242/dev.02827 17360770

[70] Bergmann C Senderek J Kupper F Schneider F Dornia C Windelen E PKHD1 mutations in autosomal recessive polycystic kidney disease (ARPKD). Hum Mutat. (2004) 23:453–63. 10.1002/humu.20029 15108277

[71] Lu H Galeano MCR Ott E Kaeslin G Kausalya PJ Kramer C. Mutations in DZIP1L, which encodes a ciliary-transition-zone protein, cause autosomal recessive polycystic kidney disease. Nat Genet. (2017) 49:1025–34. 10.1038/ng.3871 28530676 PMC5687889

[72] Perner B Englert C Bollig F. The Wilms tumor genes wt1a and wt1b control different steps during formation of the zebrafish pronephros. Dev Biol. (2007) 309:87–96. 10.1016/j.ydbio.2007.06.022 17651719

[73] Bergmann C. Educational paper: ciliopathies. Eur J Pediatr. (2012) 171:1285–300. 10.1007/s00431-011-1553-z 21898032 PMC3419833

[74] Hildebrandt F Benzing T Katsanis N. Ciliopathies. N Engl J Med. (2011) 364:1533–43. 10.1056/NEJMra1010172 21506742 PMC3640822

[75] Borgal L Habbig S Hatzold J Liebau MC Dafinger C Sacarea I. The ciliary protein nephrocystin-4 translocates the canonical Wnt regulator Jade-1 to the nucleus to negatively regulate beta-catenin signaling. J Biol Chem. (2012) 287:25370–80. 10.1074/jbc.M112.385658 22654112 PMC3408186

[76] Burckle C Gaude HM Vesque C Silbermann F Salomon R Jeanpierre C. Control of the Wnt pathways by nephrocystin-4 is required for morphogenesis of the zebrafish pronephros. Hum Mol Genet. (2011) 20:2611–27. 10.1093/hmg/ddr164 21498478

[77] French VM van de Laar IM Wessels MW Rohe C Roos-Hesselink JW Wang G. NPHP4 variants are associated with pleiotropic heart malformations. Circ Res. (2012) 110:1564–74. 10.1161/CIRCRESAHA.112.269795 22550138 PMC3916111

[78] Hoff S Halbritter J Epting D Frank V Nguyen TM van Reeuwijk J. ANKS6 is a central component of a nephronophthisis module linking NEK8 to INVS and NPHP3. Nat Genet. (2013) 45:951–6. 10.1038/ng.2681 23793029 PMC3786259

[79] Schafer T Putz M Lienkamp S Ganner A Bergbreiter A Ramachandran H. Genetic and physical interaction between the NPHP5 and NPHP6 gene products. Hum Mol Genet. (2008) 17:3655–62. 10.1093/hmg/ddn260 18723859 PMC2802281

[80] Simons M Gloy J Ganner A Bullerkotte A Bashkurov M Kronig C. Inversin, the gene product mutated in nephronophthisis type II, functions as a molecular switch between Wnt signaling pathways. Nat Genet. (2005) 37:537–43. 10.1038/ng1552 15852005 PMC3733333

[81] Slanchev K Putz M Schmitt A Kramer-Zucker A Walz G. Nephrocystin-4 is required for pronephric duct-dependent cloaca formation in zebrafish. Hum Mol Genet. (2011) 20:3119–28. 10.1093/hmg/ddr214 21596840

[82] Zhao C Malicki J. Nephrocystins and MKS proteins interact with IFT particle and facilitate transport of selected ciliary cargos. EMBO J. (2011) 30:2532–44. 10.1038/emboj.2011.165 21602787 PMC3155299

[83] Zhou W Dai J Attanasio M Hildebrandt F. Nephrocystin-3 is required for ciliary function in zebrafish embryos. Am J Physiol Renal Physiol. (2010) 299:F55–62. 10.1152/ajprenal.00043.2010 20462968 PMC2904175

[84] Adams M Simms RJ Abdelhamed Z Dawe HR Szymanska K Logan CV. A meckelin-filamin A interaction mediates ciliogenesis. Hum Mol Genet. (2012) 21:1272–86. 10.1093/hmg/ddr557 22121117 PMC3284117

[85] Duldulao NA Lee S Sun Z. Cilia localization is essential for *in vivo* functions of the Joubert syndrome protein Arl13b/Scorpion. Development (2009) 136:4033–42. 10.1242/dev.036350 19906870 PMC2778746

[86] Lessieur EM Fogerty J Gaivin RJ Song P Perkins BD. The ciliopathy gene ahi1 is required for zebrafish cone photoreceptor outer segment morphogenesis and survival. Invest Ophthalmol Vis Sci. (2017) 58:448–60. 10.1167/iovs.16-20326 28118669 PMC5270624

[87] Bachmann-Gagescu R Phelps IG Stearns G Link BA Brockerhoff SE Moens CB. The ciliopathy gene cc2d2a controls zebrafish photoreceptor outer segment development through a role in Rab8-dependent vesicle trafficking. Hum Mol Genet. (2011) 20:4041–55. 10.1093/hmg/ddr332 21816947 PMC3177654

[88] Rosenbaum JL Witman GB. Intraflagellar transport. Nat Rev Mol Cell Biol. (2002) 3:813–25. 10.1038/nrm952 12415299

[89] Amsterdam A Nissen RM Sun Z Swindell EC Farrington S Hopkins N. Identification of 315 genes essential for early zebrafish development. Proc Natl Acad Sci USA. (2004) 101:12792–7. 10.1073/pnas.0403929101 15256591 PMC516474

[90] de Vries J Yntema JL van Die CE Crama N Cornelissen EA Hamel BC Jeune syndrome: description of 13 cases and a proposal for follow-up protocol. Eur J Pediatr. (2010) 169:77–88. 10.1007/s00431-009-0991-3 19430947 PMC2776156

[91] Halbritter J Bizet AA Schmidts M Porath JD Braun DA Gee HY. Defects in the IFT-B component IFT172 cause Jeune and Mainzer-Saldino syndromes in humans. Am J Hum Genet. (2013) 93:915–25. 10.1016/j.ajhg.2013.09.012 24140113 PMC3824130

[92] Schaefer E Stoetzel C Scheidecker S Geoffroy V Prasad MK Redin C. Identification of a novel mutation confirms the implication of IFT172 (BBS20) in Bardet-Biedl syndrome. J Hum Genet. (2016) 61:447–50. 10.1038/jhg.2015.162 26763875

[93] Westhoff JH Giselbrecht S Schmidts M Schindler S Beales PL Tonshoff B. Development of an automated imaging pipeline for the analysis of the zebrafish larval kidney. PLoS ONE (2013) 8:e82137. 10.1371/journal.pone.0082137 24324758 PMC3852951

[94] Veldman MB Lin S. Zebrafish as a developmental model organism for pediatric research. Pediatr Res. (2008) 64:470–6. 10.1203/PDR.0b013e318186e609 18679162

[95] Phillips JB Westerfield M. Zebrafish models in translational research: tipping the scales toward advancements in human health. Dis Model Mech. (2014) 7:739–43. 10.1242/dmm.015545 24973743 PMC4073263

[96] Geisler R Borel N Ferg M Maier JV Strahle U. Maintenance of Zebrafish Lines at the European Zebrafish Resource Center. Zebrafish (2016) 13(Suppl. 1):S19–23. 10.1089/zeb.2015.1205 27351617 PMC4931740

[97] Sassen RW Köster R A molecular toolbox for genetic manipulation of zebrafish. Adv Genom Genet. (2015) 5:151–63. 10.2147/AGG.S57585

[98] Zhou W Boucher RC Bollig F Englert C Hildebrandt F. Characterization of mesonephric development and regeneration using transgenic zebrafish. Am J Physiol Renal Physiol. (2010) 299:F1040–7. 10.1152/ajprenal.00394.2010 20810610 PMC2980409

[99] He B Ebarasi L Hultenby K Tryggvason K Betsholtz C. Podocin-green fluorescence protein allows visualization and functional analysis of podocytes. J Am Soc Nephrol. (2011) 22:1019–23. 10.1681/ASN.2010121291 21566056 PMC3374364

[100] Seiler C Pack M. Transgenic labeling of the zebrafish pronephric duct and tubules using a promoter from the enpep gene. Gene Expr Patterns (2011) 11:118–21. 10.1016/j.gep.2010.10.002 20969977 PMC4670236

[101] Noonan HR Metelo AM Kamei CN Peterson RT Drummond IA Iliopoulos O. Loss of vhl in the zebrafish pronephros recapitulates early stages of human clear cell renal cell carcinoma. Dis Model Mech. (2016) 9:873–84. 10.1242/dmm.024380 27491085 PMC5007981

[102] Fisher S Grice EA Vinton RM Bessling SL McCallion AS. Conservation of RET regulatory function from human to zebrafish without sequence similarity. Science (2006) 312:276–9. 10.1126/science.1124070 16556802

[103] Vasilyev A Liu Y Mudumana S Mangos S Lam PY Majumdar A. Collective cell migration drives morphogenesis of the kidney nephron. PLoS Biol. (2009) 7:e9. 10.1371/journal.pbio.1000009 19127979 PMC2613420

[104] Wang Y Sun ZH Zhou L Li Z Gui JF. Grouper tshbeta promoter-driven transgenic zebrafish marks proximal kidney tubule development. PLoS ONE (2014) 9:e97806. 10.1371/journal.pone.0097806 24905828 PMC4048157

[105] Sanker S Cirio MC Vollmer LL Goldberg ND McDermott LA Hukriede NA. Development of high-content assays for kidney progenitor cell expansion in transgenic zebrafish. J Biomol Screen (2013) 18:1193–202. 10.1177/1087057113495296 23832868 PMC3830658

[106] Cianciolo Cosentino C Skrypnyk NI Brilli LL Chiba T Novitskaya T Woods C Histone deacetylase inhibitor enhances recovery after AKI. J Am Soc Nephrol. (2013) 24:943–53. 10.1681/ASN.2012111055 23620402 PMC3665399

[107] Chang MY Ma TL Hung CC Tian YC Chen YC Yang CW. Metformin inhibits cyst formation in a zebrafish model of polycystin-2 deficiency. Sci Rep. (2017) 7:7161. 10.1038/s41598-017-07300-x 28769124 PMC5541071

[108] Huang L Xiao A Wecker A McBride DA Choi SY Zhou W. A possible zebrafish model of polycystic kidney disease: knockdown of *wnt5a* causes cysts in zebrafish kidneys. J Vis Exp. (2014) e52156. 10.3791/52156 25489842 PMC4354438

[109] Blum M De Robertis EM Wallingford JB Niehrs C. Morpholinos: antisense and Sensibility. Dev Cell (2015) 35:145–9. 10.1016/j.devcel.2015.09.017 26506304

[110] Stainier DYR Raz E Lawson ND Ekker SC Burdine RD Eisen JS. Guidelines for morpholino use in zebrafish. PLoS Genet. (2017) 13:e1007000. 10.1371/journal.pgen.1007000 29049395 PMC5648102

[111] Eisen JS Smith JC. Controlling morpholino experiments: don't stop making antisense. Development (2008) 135:1735–43. 10.1242/dev.001115 18403413

[112] Kok FO Shin M Ni CW Gupta A Grosse AS van Impel A. Reverse genetic screening reveals poor correlation between morpholino-induced and mutant phenotypes in zebrafish. Dev Cell (2015) 32:97–108. 10.1016/j.devcel.2014.11.018 25533206 PMC4487878

[113] Rossi A Kontarakis Z Gerri C Nolte H Holper S Kruger M Genetic compensation induced by deleterious mutations but not gene knockdowns. Nature (2015) 524:230–3. 10.1038/nature14580 26168398

[114] Cao Y Semanchik N Lee SH Somlo S Barbano PE Coifman R. Chemical modifier screen identifies HDAC inhibitors as suppressors of PKD models. Proc Natl Acad Sci USA. (2009) 106:21819–24. 10.1073/pnas.0911987106 19966229 PMC2799791

[115] de Groh ED Swanhart LM Cosentino CC Jackson RL Dai W Kitchens CA Inhibition of histone deacetylase expands the renal progenitor cell population. J Am Soc Nephrol. (2010) 21:794–802. 10.1681/ASN.2009080851 20378823 PMC2865739

[116] Pepperkok R Ellenberg J. High-throughput fluorescence microscopy for systems biology. Nat Rev Mol Cell Biol. (2006) 7:690–6. 10.1038/nrm1979 16850035

[117] Mathias JR Saxena MT Mumm JS. Advances in zebrafish chemical screening technologies. Future Med Chem. (2012) 4:1811–22. 10.4155/fmc.12.115 23043478 PMC3566566

[118] Yanik MF Rohde CB Pardo-Martin C. Technologies for micromanipulating, imaging, and phenotyping small invertebrates and vertebrates. Annu Rev Biomed Eng. (2011) 13:185–217. 10.1146/annurev-bioeng-071910-124703 21756142

[119] Burns CG Milan DJ Grande EJ Rottbauer W MacRae CA Fishman MC. High-throughput assay for small molecules that modulate zebrafish embryonic heart rate. Nat Chem Biol. (2005) 1:263–4. 10.1038/nchembio732 16408054

[120] Kokel D Bryan J Laggner C White R Cheung CY Mateus R. Rapid behavior-based identification of neuroactive small molecules in the zebrafish. Nat Chem Biol. (2010) 6:231–7. 10.1038/nchembio.307 20081854 PMC2834185

[121] Lin S Zhao Y Xia T Meng H Ji Z Liu R. High content screening in zebrafish speeds up hazard ranking of transition metal oxide nanoparticles. ACS Nano (2011) 5:7284–95. 10.1021/nn202116p 21851096 PMC4136441

[122] Molina G Vogt A Bakan A Dai W Queiroz de Oliveira P Znosko W. Zebrafish chemical screening reveals an inhibitor of Dusp6 that expands cardiac cell lineages. Nat Chem Biol. (2009) 5:680–7. 10.1038/nchembio.190 19578332 PMC2771339

[123] Pylatiuk C Sanchez D Mikut R Alshut R Reischl M Hirth S. Automatic zebrafish heartbeat detection and analysis for zebrafish embryos. Zebrafish (2014) 11:379–83. 10.1089/zeb.2014.1002 25003305 PMC4108935

[124] Zon LI Peterson RT. *In vivo* drug discovery in the zebrafish. Nat Rev Drug Discov. (2005) 4:35–44. 10.1038/nrd1606 15688071

[125] Wittbrodt JN Liebel U Gehrig J. Generation of orientation tools for automated zebrafish screening assays using desktop 3D printing. BMC Biotechnol. (2014) 14:36. 10.1186/1472-6750-14-36 24886511 PMC4021294

[126] Peravali R Gehrig J Giselbrecht S Lutjohann DS Hadzhiev Y Muller F. Automated feature detection and imaging for high-resolution screening of zebrafish embryos. Biotechniques (2011) 50:319–24. 10.2144/000113669 21548893

[127] Pardo-Martin C Chang TY Koo BK Gilleland CL Wasserman SC Yanik MF. High-throughput *in vivo* vertebrate screening. Nat Methods (2010) 7:634–6. 10.1038/nmeth.1481 20639868 PMC2941625

[128] Westhoff JH Steenbergen PJ Wagner J Tönshoff B Liebel U Gehrig J *In vivo* high content screening in zebrafish to score developmental nephrotoxicity of approved drugs [abstract]. In: 10th European Zebrafish Meeting. Budapest (2017) P-II-157.

[129] Pandey G Westhoff JH Schaefer F Gehrig J An automated high content screening platform to identify cystic kidney disease-modifying substances in zebrafish [abstract]. In: 55th European Renal Association & European Dialysis and Transplant Association. Copenhagen (2018) SuO018.

[130] Pardo-Martin C Allalou A Medina J Eimon PM Wahlby C Fatih Yanik M. High-throughput hyperdimensional vertebrate phenotyping. Nat Commun. (2013) 4:1467. 10.1038/ncomms2475 23403568 PMC3573763

[131] Peterson RT Fishman MC. Designing zebrafish chemical screens. Methods Cell Biol. (2011) 105:525–41. 10.1016/B978-0-12-381320-6.00023-0 21951546

[132] Conrad C Wunsche A Tan TH Bulkescher J Sieckmann F Verissimo F. Micropilot: automation of fluorescence microscopy-based imaging for systems biology. Nat Methods (2011) 8:246–9. 10.1038/nmeth.1558 21258339 PMC3086017

[133] Gunkel M Eberle JP Erfle H. Fluorescence-based high-throughput and targeted image acquisition and analysis for phenotypic screening. Methods Mol Biol. (2017) 1563:269–80. 10.1007/978-1-4939-6810-7_17 28324614

[134] Tischer C Hilsenstein V Hanson K Pepperkok R. Adaptive fluorescence microscopy by online feedback image analysis. Methods Cell Biol. (2014) 123:489–503. 10.1016/B978-0-12-420138-5.00026-4 24974044

[135] Mikut R Dickmeis T Driever W Geurts P Hamprecht FA Kausler BX. Automated processing of zebrafish imaging data: a survey. Zebrafish (2013) 10:401–21. 10.1089/zeb.2013.0886 23758125 PMC3760023

